# Multi-Metric Assessment of Liquid Chromatography–Mass Spectrometry Methods for Mycotoxin Determination in Human Urine: Advancing Sustainable, Practical, and Innovative Analytical Practices

**DOI:** 10.3390/ph19081231

**Published:** 2026-08-05

**Authors:** Abdulmalik M. Alqarni, Heba Shaaban, Ahmed Mostafa, Khalid Alenazi, Owais Dahlawi, Essam M. Hafez, Hassan A. Alqarni, Mohammad A. Alrofaidi, Mohammed Alqarni

**Affiliations:** 1 Department of Pharmaceutical Chemistry, College of Pharmacy, Imam Abdulrahman Bin Faisal University, King Faisal Road, P.O. Box 1982, Dammam 31441, Saudi Arabiaammostafa@iau.edu.sa (A.M.); 2 College of Pharmacy, Imam Abdulrahman Bin Faisal University, King Faisal Road, P.O. Box 1982, Dammam 31441, Saudi Arabia; 3 Department of Forensic Medicine and Clinical Toxicology, Faculty of Medicine, Minia University, Minia 61519, Egypt; 4 College of Medicine, Imam Abdulrahman Bin Faisal University, Dammam 31441, Saudi Arabia; 5 Department of Pharmaceutical Chemistry, Faculty of Pharmacy, Al-Baha University, Al-Baha P.O. Box 1988, Saudi Arabia; 6 Department of Pharmaceutical Chemistry, College of Pharmacy, Taif University, P.O. Box 11099, Taif 21944, Saudi Arabia

**Keywords:** mycotoxins, green analytical chemistry, human biomonitoring, AGREE, IMOGI, pharmaceutical analysis

## Abstract

**Background/Objectives:** Human biomonitoring of mycotoxins is important for assessing exposure and its associated health risks. Modern analytical chemistry aims to enhance sustainability, practicality, and modernization of analytical methods. Therefore, developing sensitive, selective, cost-effective, and environmentally friendly methods is highly desirable. This study aimed at evaluating liquid chromatographic methods reported for the determination of mycotoxins in human urine, identifying their strengths, limitations, and future development needs and providing guidance for selecting the most appropriate methods. **Methods:** Twelve liquid chromatographic methods for mycotoxin determination in urine were selected. The methods were evaluated in terms of environmental sustainability, practical applicability, and innovation. Five complementary metrics, namely the Analytical GREEnness Metric, Modified Green Analytical Procedure Index, Click Analytical Chemistry Index, Blue Applicability Grade Index, and Innovative Method Orange Gradient Index, were utilized. **Results:** Most of the evaluated methods exhibited acceptable levels of eco-friendliness and moderate to good practical applicability, but limited innovation. Within the scope of the selected multi-criteria assessment framework, method 5 achieved the highest overall score owing to its multianalyte capability, efficient sample preparation, reduced sample and solvent consumption, and favorable sustainability, practicality, and innovation characteristics. **Conclusions:** The findings highlight the need for more practical and technologically advanced analytical methods that align with the principles of green and modern analytical chemistry principles, thereby supporting more sustainable and efficient biomonitoring of mycotoxins.

## 1. Introduction

Mycotoxins are a diverse group of toxic low-molecular-weight compounds produced by fungi that contaminate food and feed [1]. Exposure to mycotoxins causes adverse health effects, such as carcinogenicity, mutagenicity, hepatotoxicity, nephrotoxicity, neurotoxicity, immunotoxicity, endocrine-disrupting effects, inflammation, and gastrointestinal dysfunction [2,3,4,5]. Mycotoxins are considered the most common foodborne pollutants and recognized as emerging contaminants of concern [6,7].

Human biomonitoring provides valuable information on exposure to mycotoxins by enabling the direct assessment of internal dosage [8]. Urine is considered the preferred biological matrix for this purpose because sample collection is non-invasive and urinary excretion represents a major elimination pathway for several mycotoxins and their metabolites. Consequently, the analysis of urine samples offers significant advantages for exposure assessment and provides a more reliable estimate of mycotoxin intake [9,10].

Different analytical methods have been developed for the determination of mycotoxins in human urine for biomonitoring and exposure assessment purposes, employing a variety of sample preparation strategies and chromatographic separation techniques [11,12]. However, many conventional methods rely heavily on hazardous organic solvents and reagents that may pose risks to both human health and the environment [13,14]. In response to these concerns, the principles of green analytical chemistry (GAC) have been introduced to minimize the environmental footprint of analytical procedures and improve their safety for both practitioners and ecosystems [15].

For greening analytical methods, different strategies were introduced, including substituting conventional harmful solvents with more benign biodegradable ones, reducing solvent consumption and minimizing waste generation [16]. For reducing solvent consumption and shortening analysis time, different approaches were implemented, such as using analytical columns packed with fused core and sub-2 μm particles, and high-temperature liquid chromatography [17,18,19,20]. Analytical methods that are simple, cost-effective, eco-friendly and practical are required for routine analysis [21]. The introduction of analytical chemistry metrics facilitates the assessment of analytical methods in terms of different perspectives, including environmental impact, practicality and innovation, thereby enhancing the selection of balanced methods [22]. These metrics include the Analytical GREEnness Metric (AGREE) [23] and the Modified Green Analytical Procedure Index (MoGAPI) [24] for evaluating environmental sustainability, the Blue Applicability Grade Index (BAGI) [25] and the Click Analytical Chemistry Index (CACI) [26] for assessing the practical applicability, and the recently introduced Innovative Method Orange Gradient Index (IMOGI) [27] for assessing the degree of innovation and modernization of analytical procedures. The combined application of these complementary tools offers deeper insight into the strengths and limitations of analytical methods than can be obtained using a single evaluation criterion [28].

Because mycotoxins are typically encountered at trace levels in complex biological matrices, reliable human biomonitoring requires analytical methods that combine high sensitivity and selectivity with compliance with GAC principles and suitability for routine application. Therefore, the aim of the present study was to comprehensively evaluate selected liquid chromatographic methods reported for the determination of mycotoxins in human urine with respect to their environmental sustainability, practical applicability, and degree of innovation using multiple assessment tools. In addition, the study sought to identify the major factors governing method performance, highlight current limitations, and provide guidance for the selection and future development of more balanced analytical methods. To the best of our knowledge, this is the first study to provide a holistic, multi-metric evaluation of liquid chromatographic methods for mycotoxin determination in human urine by simultaneously considering sustainability, practicality, and innovation.

## 2. Results and Discussion

### 2.1. Assessment of the Environmental Impact of the Reported Methods

**Figure 1:**
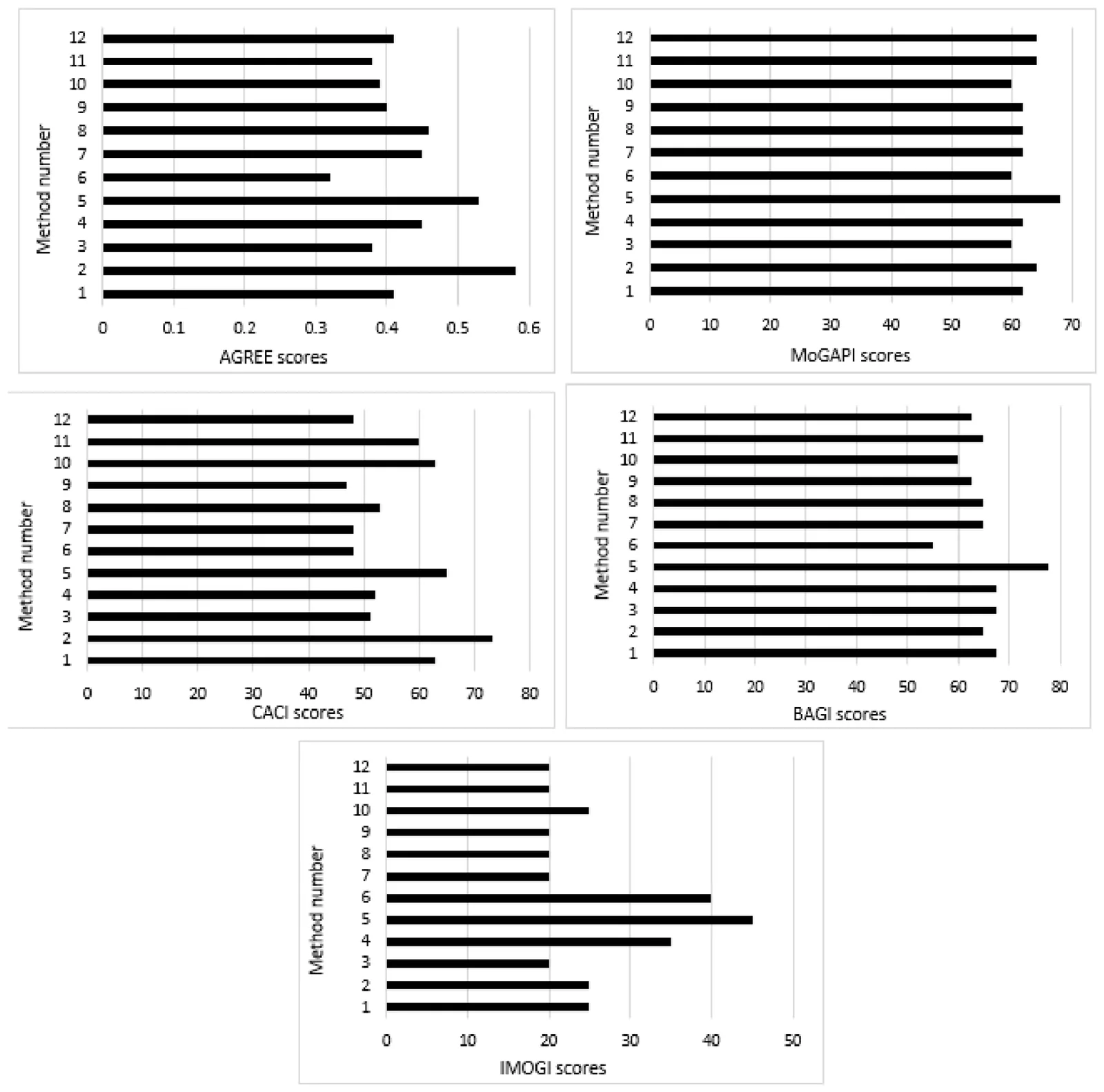
Final scores correspond to the analytical methods evaluated for the determination of mycotoxins in human urine using AGREE, MoGAPI, CACI, BAGI and IMOGI assessment tools.

The environmental sustainability of the twelve selected liquid chromatographic methods reported for mycotoxin determination in human urine was assessed using AGREE and MoGAPI assessment tools. The utilization of both metrics provides a comprehensive insight on the greenness profile of the investigated methods by considering multiple aspects of GAC. As illustrated in Figure 1, the environmental assessment revealed a relatively narrow distribution of scores, indicating that although the investigated methods differ in their degree of compliance with GAC principles, most have incorporated several environmentally conscious analytical practices. However, none of the evaluated methods achieved exceptionally high greenness scores, highlighting that further improvements in environmental sustainability remain necessary despite recent advances in chromatographic analysis.

The calculated AGREE scores ranged from 0.32 to 0.58, indicating moderate variability in compliance with GAC principles. Among the investigated procedures, method 2 achieved the highest AGREE score (0.58), suggesting the most favorable environmental profile. This performance can be attributed to the use of microscale sample preparation involving only 100 μL of urine combined with online SPE-UHPLC-MS/MS analysis (Table 1). Moreover, the simultaneous determination of eleven mycotoxins in a single run is in accordance with GAC principles, as it maximizes analytical information while minimizing sample consumption, solvent usage, waste generation, and energy requirements. The high AGREE score further demonstrates that miniaturized analytical workflows and multianalyte capability substantially improve the environmental sustainability of chromatographic methods by maximizing analytical efficiency while minimizing resource consumption. Method 5 also exhibited relatively high greenness (AGREE = 0.53). Its favorable performance is mainly associated with the use of a dilute-and-shoot approach, which considerably simplifies sample preparation and reduces reagent consumption while maintaining high analytical sensitivity. This finding demonstrates that simplified analytical workflows can simultaneously improve operational efficiency and reduce environmental burden without compromising analytical performance. In contrast, method 6 showed the lowest AGREE score (0.32), indicating comparatively lower environmental sustainability. This reduced greenness can be attributed to the relatively large sample volume requirement (30 mL), multistep sample preparation, and the determination of only five analytes, which limits the amount of analytical information obtained per analysis. Additionally, the method demonstrated moderate sensitivity and relies on unreadily available chemicals and reagents for the extraction, leading to low compliance with the GAC principles. These observations indicate that environmental sustainability is influenced not only by solvent consumption but also by overall workflow design, including sample throughput, extraction complexity, and analytical efficiency. Consequently, methods requiring extensive sample handling and providing limited analytical information per analysis tend to exhibit reduced greenness.

To further evaluate the environmental impact of the investigated methods, the MoGAPI metric was applied. The calculated MoGAPI scores ranged from 60 to 68, indicating moderate to good environmental performance for all evaluated procedures. Method 5 achieved the highest MoGAPI score (68), reflecting the environmental benefits associated with the simplified dilute-and-shoot strategy and efficient resource utilization. Methods 2 and 12 followed with scores of 64, further confirming their favorable environmental profiles. In contrast, methods 3, 6, 10, and 11 exhibited the lowest MoGAPI scores (60). Nevertheless, the relatively narrow distribution of MoGAPI values indicates that none of the investigated methods exhibited markedly poor environmental performance. As shown in Figure 1, the small variation in MoGAPI scores suggests that most recently developed methods have adopted at least some green analytical practices, although the extent of implementation varies among the investigated procedures. Good agreement was observed between the AGREE and MoGAPI evaluations. Among all evaluated methods, methods 2 and 5 are considered the most environmentally friendly methods, which is attributed to the minimized solvent consumption, low sample volume, high sensitivity, analysis of multianalytes and simplified extraction, all of which are closely aligned with GAC principles. Although AGREE and MoGAPI employ different assessment criteria and weighting strategies, both metrics consistently identified methods 2 and 5 as the most environmentally sustainable procedures. This agreement strengthens confidence in the environmental assessment and indicates that the observed ranking is not dependent on a single evaluation tool. Minor differences between the two metrics are primarily attributed to differences in their scoring methodologies rather than contradictory evaluations of the investigated methods. Conversely, large sample volumes, labor-intensive workflows, limited analyte coverage, and increased resource requirements adversely affect environmental performance. Interestingly, the results indicate that multianalyte capability represents an important contributor to environmental sustainability. Methods capable of simultaneously determining a larger number of mycotoxins maximize the analytical information obtained from a single analysis, thereby reducing sample consumption, solvent usage, energy requirements, and waste generation on a per-analyte basis. This finding supports the current trend toward comprehensive multiresidue analytical methods for human biomonitoring applications. Overall, the environmental assessment demonstrates that improvements in chromatographic instrumentation alone are insufficient to maximize method greenness. Instead, simplified analytical workflows, miniaturized sample preparation, reduced resource consumption, and multianalyte determination collectively represent the primary factors driving environmentally sustainable analytical methods. Therefore, future method development should adopt an integrated design strategy that simultaneously considers analytical performance and environmental sustainability.

The scores and pictograms for the reported methods are presented in Table 2.

### 2.2. Assessment of the Applicability of the Reported Methods

The applicability of the investigated methods was evaluated using the CACI and BAGI metrics. The CACI scores ranged from 47 to 73, which indicates a considerable variation in the practicality levels of the evaluated procedures. As shown in Figure 1, the relatively wide distribution of CACI scores demonstrates that practical applicability varies more substantially among the investigated methods than environmental sustainability. This observation suggests that while many methods have adopted greener analytical practices, their operational feasibility is strongly influenced by workflow design, sample preparation complexity, and analytical throughput. Consequently, practicality remains one of the major factors differentiating current chromatographic methods for mycotoxin determination in human urine. Among all methods, method 2 showed the highest CACI score of 73, reflecting superior operational feasibility. The driving forces for this favorable performance are the use of microscale sample volume (100 μL), high analytical sensitivity, and the simultaneous determination of eleven mycotoxins in a single run, which maximizes analytical information and improves laboratory efficiency. Furthermore, the method relied on readily available solvents and reagents such as acetonitrile, ammonium acetate, and formic acid. Also, the integration of online SPE further enhances the practicality of the method by reducing analyst intervention, improving reproducibility, and increasing sample throughput, making it particularly suitable for routine biomonitoring laboratories handling large numbers of samples. Method 5 also demonstrated high practicality (CACI score is 65). This favorable score is mainly attributed to the use of a dilute-and-shoot approach, which involves a low-cost and straightforward sample preparation procedure. Furthermore, the method requires only 800 μL of urine, employs commonly available reagents such as acetonitrile and formic acid, exhibits high analytical sensitivity, and enables the simultaneous determination of twelve mycotoxins. The consistently high ranking of method 5 demonstrates that simplified analytical workflows can substantially improve routine applicability without compromising analytical performance, highlighting workflow simplification as a key strategy for developing efficient biomonitoring methods. Although method 10 is likewise based on a dilute-and-shoot strategy and requires an even smaller sample volume (100 μL) while maintaining high sensitivity, its practicality scores were comparatively lower. This difference may be associated with the analysis of few analytes (only three), hence limiting analytical throughput. Additionally, this method involves the use of a larger variety of organic solvents, resulting in increased resource requirements. This comparison illustrates that practical applicability depends on multiple complementary factors rather than a single parameter. Although miniaturized sample preparation contributes positively to practicality, its overall benefit may be offset by limited analyte coverage or increased operational requirements. Therefore, comprehensive multianalyte determination represents an important factor for improving laboratory productivity and cost-effectiveness. In contrast, methods 6, 7, 9, and 12 demonstrated the lowest CACI scores (47–48), indicating comparatively lower applicability. These methods generally relied on more labor-intensive workflows, greater manual intervention, or lower analytical throughput, thereby increasing the time and resources required for routine implementation. Such characteristics reduce their suitability for high-throughput biomonitoring programs, where operational efficiency is essential.

For further assessment of the methods, the BAGI metric was employed. The BAGI scores were in the range of 55 to 77.5, reflecting moderate to excellent applicability for most of the evaluated methods. As illustrated in Figure 1, most methods achieved moderate to high BAGI scores, indicating that recent analytical developments have generally considered operational applicability alongside analytical performance. However, the variability in BAGI values also demonstrates that considerable opportunities remain to simplify analytical workflows and improve routine implementation. Again, method 5 achieved the highest BAGI score (77.5), highlighting that the use of a simple and inexpensive dilute-and-shoot sample preparation approach, readily available reagents, a shortened analytical workflow and simultaneous multi-analyte analysis were the main factors contributing to its superior practicality and suitability for routine laboratory applications. Methods 1, 3, and 4 also demonstrated excellent applicability, with BAGI values of 67.6, 67.5, and 67.5, respectively. In contrast, method 6 exhibited the lowest BAGI score (55), indicating reduced applicability for routine analysis. The low performance is mainly due to the relatively large sample volume requirement (30 mL) and the determination of only five analytes, which reduce analytical throughput. Overall, good agreement was observed between the CACI and BAGI evaluations, with methods 2 and 5 ranked as the most practical and applicable procedures. Although CACI and BAGI evaluate practicality using different criteria, both metrics consistently identified the same methods as the most operationally feasible, supporting the robustness of the comparative assessment. The consistency between the two independent metrics indicates that the observed rankings reflect genuine differences in analytical workflow design rather than variations in the scoring methodology. The results indicate that high practicality is primarily driven by miniaturized sample requirements, simplified and low-cost sample preparation, the use of readily available reagents, high analytical sensitivity, and the simultaneous determination of multiple analytes. Notably, multianalyte capability emerged as one of the major contributors to method applicability. Determining a larger number of mycotoxins in a single analytical run reduces instrument time, sample consumption, reagent usage, and overall analytical cost on a per-analyte basis, thereby improving laboratory efficiency and supporting large-scale human biomonitoring studies. These features reduce operational complexity, thereby making the methods particularly attractive for routine biomonitoring applications. Conversely, extensive sample preparation, increased procedural complexity, and lower analytical efficiency adversely affect method applicability. Overall, the applicability assessment demonstrates that the routine implementation of chromatographic methods depends not only on analytical performance but also on the integration of simplified sample preparation, high analytical throughput, and efficient resource utilization. Consequently, future analytical method development should focus on designing workflows that simultaneously maximize operational simplicity, multianalyte capability, and analytical performance to facilitate their adoption in routine biomonitoring laboratories.

### 2.3. Assessment of the Methods’ Innovation

The level of innovation and modernization of the evaluated methods was assessed using the recently introduced IMOGI tool. The calculated scores of IMOGI ranged from 20 to 45, which indicates variance in the degrees of innovation and advancement of the investigated methods. According to the IMOGI classification, methods scoring below 30 are considered low in innovation, those scoring between 30 and 50 are acceptably innovative, whereas methods scoring above 50 are classified as highly innovative. As illustrated in Figure 1, the majority of the evaluated methods clustered within the low-innovation category, whereas only three methods achieved acceptable innovation scores. This distribution suggests that, despite the continuous improvements in analytical performance in recent years, methodological innovation has advanced at a comparatively slower pace in chromatographic methods for urinary mycotoxin analysis. IMOGI assesses methodological innovation rather than analytical performance alone. The assessment is based on ten complementary criteria that examine different aspects of innovation, including automation, miniaturization, advanced instrumentation, integration of digital tools or artificial intelligence, adaptability, introduction of novel analytical concepts, utilization of novel materials or reagents, improvements in analytical efficiency and throughput, interdisciplinary integration, and implementation of green and white analytical chemistry principles. Consequently, the final IMOGI score reflects the cumulative contribution of all innovation criteria rather than excellence in a single aspect of the analytical method.

Method 5 achieved the highest IMOGI score (45), placing it within the acceptably innovative category. Its favorable score is attributed to the fulfillment of several IMOGI criteria rather than a single innovative feature. Although the method did not introduce a completely new analytical concept, it combined high analytical performance, high-throughput multianalyte determination, simplified dilute-and-shoot sample preparation, reduced sample and solvent consumption, and explicit implementation of GAC principles. This integration of multiple methodological improvements resulted in the highest overall innovation score among the evaluated methods. Method 6 also demonstrated acceptable innovation (IMOGI = 40), primarily due to the introduction of a novel dispersive solid-phase extraction approach based on magnetic carbon nanotube composites. The use of magnetic carbon nanotube composites represents a genuine methodological innovation and fully satisfies the IMOGI criterion related to the introduction of a novel analytical concept and the utilization of innovative materials. However, despite this technological advancement, the method did not substantially improve analytical throughput compared with existing methods, and did not explicitly incorporate GAC principles. Consequently, although method 6 introduced an innovative extraction technology, its overall IMOGI score was slightly lower than that of method 5.

In contrast, the majority of the investigated methods exhibited low innovation, with IMOGI scores ranging from 20 to 25. In particular, methods 3, 7, 8, 9, 11, and 12 obtained the lowest score (20), indicating a greater reliance on conventional analytical workflows and limited integration of modern extraction materials, alternative sampling strategies, or green analytical concepts. These methods generally focused on optimizing established analytical procedures rather than introducing substantial methodological advances across multiple innovation criteria. Notably, although all the evaluated methods employed LC-MS/MS for detection, they exhibited different innovation levels because IMOGI evaluates innovation using multiple predefined methodological criteria rather than assigning scores based solely on the analytical instrumentation. Consequently, excellent analytical sensitivity or the use of advanced instrumentation alone is insufficient to achieve a high innovation score. Only three methods were classified as acceptably innovative, whereas the remaining procedures fell within the low-innovation category. None of the evaluated methods demonstrated a high level of innovation. Overall, the results indicate that innovation in chromatographic methods for determination of mycotoxins in human urine remains largely incremental rather than transformative. Future developments should therefore focus on integrating multiple innovation elements within a single analytical workflow, including environmentally benign sample preparation, automation, digital technologies, and comprehensive multianalyte determination, thereby simultaneously enhancing sustainability, practicality, and methodological innovation.

### 2.4. Overall Assessment and Future Perspectives

The present study provides more than a comparison of published methods by identifying the methodological characteristics that collectively govern environmental sustainability, practical applicability, and innovation. The integrated multi-metric evaluation demonstrated that analytical performance alone is insufficient for selecting the most appropriate analytical method. Instead, optimal methodologies should simultaneously achieve high analytical performance while minimizing environmental impact, simplifying analytical workflows, maximizing analytical throughput, and incorporating innovative methodological features. These findings provide valuable insights for the future development of sustainable, practical, and innovative analytical methods and demonstrate the value of integrating complementary assessment metrics to support evidence-based method selection. In addition, the present assessment offers guidance for selecting analytical methods according to the intended biomonitoring application. Rather than recommending a single method for all situations, the comparative assessment indicates that method selection should be guided by the target mycotoxins, analytical objectives, required analyte coverage, available laboratory resources, and environmental considerations. For routine biomonitoring requiring the simultaneous determination of multiple mycotoxins, methods combining simplified sample preparation, high throughput, and broad multianalyte capability are preferred. When minimizing the environmental impact of the analysis is a priority, analytical methods employing microscale sample preparation, reduced sample and solvent consumption, simplified workflows, and environmentally benign reagents should be selected whenever possible, as these approaches minimize waste generation and resource consumption while maintaining reliable analytical performance. Conversely, studies targeting specific or emerging mycotoxins may benefit from specialized extraction strategies or novel analytical approaches when additional selectivity or methodological innovation is required. Therefore, the present assessment provides a decision-support framework that enables researchers to select the most appropriate analytical method according to analytical requirements while balancing analytical performance, environmental sustainability, practical applicability, and methodological innovation.

The overall assessment indicates that current methodologies for determination of mycotoxins in human urine have achieved moderate progress toward sustainability and modernization. However, most approaches still rely on conventional workflows and exhibit only incremental improvements. The predominance of moderate levels of greenness, practicality and innovation suggests that significant opportunities remain for methodological advancement. The comparative assessment further identified the major methodological factors influencing overall method performance, including the dependence on hazardous organic solvents, labor-intensive sample preparation, high resource demands, restricted analyte coverage, and insufficient incorporation of emerging analytical concepts. Therefore, future developments should not only focus on analytical performance but also consider environmental and operational aspects. Future research should prioritize the development of analytical workflows that further reduce sample and solvent consumption while increasing analyte coverage and throughput. Greater emphasis should be placed on miniaturized and automated sample preparation techniques, the use of environmentally benign reagents, and simplified procedures that minimize manual intervention. Furthermore, greater integration of digital technologies, automation, and innovative sample preparation strategies should be encouraged to accelerate methodological modernization. Such advancements would improve the balance between sustainability, practicality, and innovation, ultimately enabling more efficient and reliable human biomonitoring.

Despite the comprehensive nature of the present multi-metric assessment, it should be noted that the evaluation was based exclusively on methodological information reported in the published literature. Therefore, the accuracy of the assessment depends on the completeness and quality of the reported experimental details. Methods lacking sufficient information could not be included, which may have limited the number of eligible studies. In addition, the assessment tools evaluate predefined criteria related to environmental sustainability, practical applicability, and methodological innovation, but they cannot fully account for laboratory-specific factors such as instrument availability, operator expertise, implementation costs, or local infrastructure.

## 3. Materials and Methods

### 3.1. Literature Search and Method Selection

**Figure 2:**
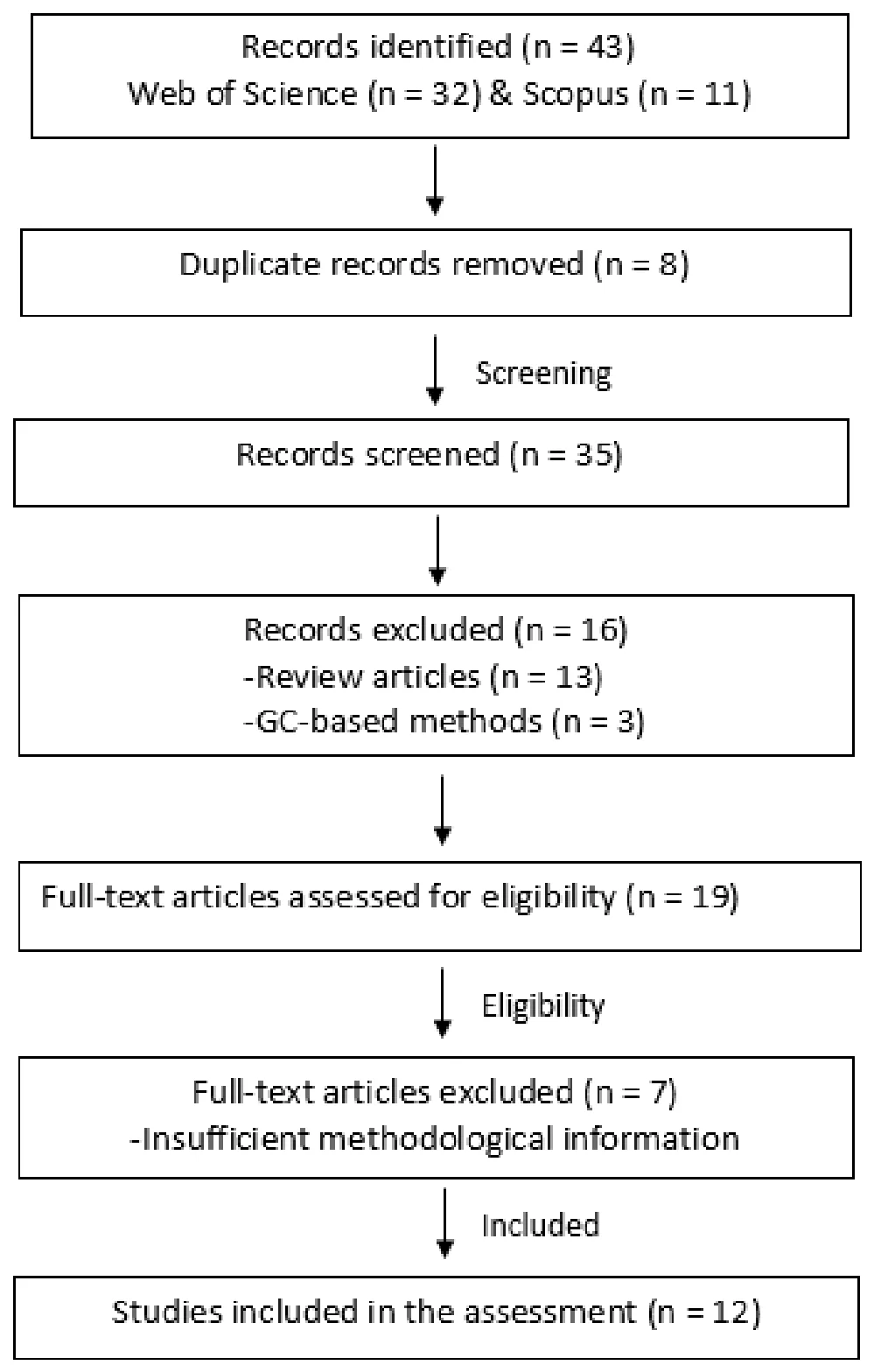
Flow diagram illustrating the literature search and study selection process for identifying eligible studies included in the comparative assessment.

A structured literature search was conducted to identify published liquid chromatographic methods for the determination of mycotoxins in human urine. The search was performed using the Scopus and Web of Science databases and was limited to studies published between 2020 and 2026. This publication period was selected to ensure that the assessment reflects recent advances in chromatographic instrumentation, sample preparation strategies, and the principles of sustainable and modern analytical chemistry. The literature search was conducted using combinations of relevant keywords, including mycotoxins, human urine, biomonitoring, liquid chromatography, HPLC, UHPLC, LC-MS, and LC-MS/MS. The study selection process is summarized in Figure 2. The retrieved articles were screened according to predefined eligibility criteria. Studies were included if they (i) reported an original liquid chromatography-based analytical method for the determination of one or more mycotoxins in human urine; (ii) provided sufficient experimental details to enable evaluation using the selected greenness, practicality, and innovation assessment metrics; and (iii) were published in peer-reviewed journals in English. Studies were excluded if they (i) investigated matrices other than human urine; (ii) employed analytical techniques other than liquid chromatography; or (iii) were review articles, conference abstracts, or book chapters. Because the selected assessment tools (AGREE, MoGAPI, CACI, BAGI, and IMOGI) require comprehensive methodological information to ensure a reliable and consistent evaluation, studies lacking sufficient experimental details were excluded, as incomplete methodological information would not allow for accurate assessment using these metrics. All retrieved articles were further assessed to ensure their relevance and consistency with the scope of this work. Following the screening process, twelve chromatographic methods fulfilled the predefined eligibility criteria and were selected for comparative assessment. Although the literature search included all liquid chromatographic methods, all studies meeting the predefined eligibility criteria employed LC-MS/MS for mycotoxin determination in human urine. For each selected method, key analytical characteristics, including the sample preparation protocol, sample volume, solvent type and consumption, chromatographic separation and detection systems, analysis time, number of analytes determined, and analytical performance characteristics, were extracted from the corresponding publications. The collected information served as the basis for the comparative multi-criteria evaluation carried out using the selected assessment tools.

### 3.2. Assessment Tools

The selected chromatographic methods were comprehensively evaluated using five complementary assessment tools to assess their environmental sustainability, practical applicability, and methodological innovation. The assessment tools included the Analytical GREEnness Metric (AGREE), Modified Green Analytical Procedure Index (MoGAPI), Click Analytical Chemistry Index (CACI), Blue Applicability Grade Index (BAGI), and Innovative Method Orange Gradient Index (IMOGI).

Environmental aspects were examined using AGREE [23] and MoGAPI [24]. AGREE evaluates analytical procedures according to the 12 principles of GAC and provides a holistic evaluation of the environmental impact of analytical methods. AGREE considers sample amount, reagent toxicity, energy consumption, waste generation, automation, miniaturization, and multianalyte capability. The results are presented as a circular pictogram and an overall score ranging from 0 to 1, where higher values indicate better environmental performance [23]. The circular pictogram further enables rapid visualization of the contribution of each GAC principle to the final score.

In contrast, MoGAPI focuses on the environmental impact of the entire analytical workflow, including sample collection, preservation, transportation, sample preparation, reagents, instrumentation, occupational hazards, and waste production. The results are presented as a pictogram with a score ranging from 0 to 100 [24]. AGREE provides a principle-based holistic evaluation, whereas MoGAPI places greater emphasis on identifying environmentally unfavorable stages throughout the analytical workflow. Consequently, the combined application of AGREE and MoGAPI provides a more comprehensive assessment of method greenness than either metric alone, allowing both overall environmental performance and specific environmental weaknesses to be identified.

For assessing the practical applicability of the reported methods, BAGI [25] and CACI [26] metrics were used. Although both metrics assess the suitability of analytical methods for routine laboratory implementation, they emphasize different aspects of method applicability and therefore provide complementary rather than redundant information.

BAGI assessment is based on ten criteria, including analysis type, number of analytes, sample throughput, reagent and material requirements, instrumentation, simultaneous sample treatment, preconcentration needs, automation, sample preparation, and sample amount. Consequently, BAGI provides a broad assessment of the operational feasibility of a method from the perspective of routine laboratory implementation. BAGI scores range from 0 to 100, with higher values indicating greater applicability [25]. The visual star-shaped pictogram facilitates rapid identification of the strengths and weaknesses of individual analytical procedures.

CACI evaluates practicality through ten parameters, including sample size, sample preparation requirements, preparation time, feasibility, instrument availability, analytical cost, portability, automation, and total analysis time. Unlike BAGI, CACI explicitly incorporates analytical sensitivity as one of its assessment criteria, thereby recognizing methods capable of achieving reliable quantification while maintaining practical laboratory operation. The overall score also ranges from 0 to 100, where higher scores reflect better practicality and ease of implementation [26]. The combined use of CACI and BAGI therefore provides a more balanced evaluation of method applicability by simultaneously considering analytical capability and practical laboratory implementation. This complementary assessment enables a more informed selection of analytical methods according to the intended application.

IMOGI complements the previous four metrics by evaluating methodological innovation rather than environmental sustainability or practical applicability [27]. The index considers ten attributes related to methodological novelty, technological advancement, automation, miniaturization, innovative materials, sustainability, and integration of emerging concepts. Unlike conventional analytical performance parameters, IMOGI does not assign higher scores solely because a method achieves lower detection limits or employs advanced instrumentation. Instead, it assesses whether the method introduces meaningful methodological advances beyond established analytical practices. IMOGI generates a cumulative score between 0 and 100, with scores below 30 indicating low innovation, scores between 30 and 50 corresponding to acceptable innovation, and scores above 50 representing highly innovative analytical methods.

The combined use of AGREE, MoGAPI, CACI, BAGI, and IMOGI therefore provides a multidimensional assessment framework that simultaneously evaluates environmental sustainability, practical applicability, and methodological innovation. Together, these complementary metrics enable a more balanced and evidence-based comparison of analytical methods than could be achieved using any individual assessment tool alone.

The principal characteristics of the selected liquid chromatographic procedures for mycotoxin determination in human urine are summarized in Table 1, while the graphical outputs generated by AGREE, MoGAPI, BAGI, CACI, and IMOGI are compiled in Table 2. The information obtained from these complementary metrics formed the basis for the subsequent comparative assessment of environmental sustainability, practical applicability, and methodological innovation.

## 4. Conclusions

The present study demonstrated the usefulness of a multi-criteria approach for the comparative evaluation of liquid chromatographic methods used for mycotoxin determination in human urine. The results revealed marked differences among the investigated procedures and showed that no single metric can adequately reflect overall method quality. Among all evaluated methods, method 5 demonstrated the most balanced performance in terms of environmental sustainability, applicability, and innovation. This study pointed out the shortcoming of the existing analytical procedures for the determination of mycotoxins in human urine and emphasized the necessity of developing more practical and advanced approaches that adhere to green and modern analytical chemistry principles. Environmental, practical, and innovation aspects should be taken into consideration during method selection and development, thereby advancing analytical strategies for human biomonitoring.

## Figures and Tables

**Table 1:** Chromatographic conditions for LC methods reported for the analysis of mycotoxins in human urine.

No.	Analytes	Separation and Detection Method	Extraction Method	Solvents and Reagents Used Throughout the Analytical Procedure	LOD	Sample Volume	Ref.
1	Enniatin A, B, A1, and B1; beauvericine; aflatoxin B1, B2, G1 and G2; ochratoxin	LC-Q-TOF-MS	QuEChERS	Acetonitrile, formic acid, MgSO_4_	0.1–1.5 ng/mL	1 mL	[29]
2	Aflatoxin, altenuene, alternariol monomethyl ether, alternariol, citrinin and its metabolite dihydrocitrinone, fumonisin B1, ochratoxin, zearalenone, α- and β-zearalenol	UHPLC-MS/MS	online SPE	Acetonitrile, ammonium acetate, formic acid	0.0036–0.27 ng/mL	100 µL	[30]
3	Fumonisins, ochratoxins, alternaria and emerging fusarium mycotoxins (fumonisin B1, B2, and B3; hydrolyzed fumonisin B1 and B2; ochratoxin A, B, alpha; alternariol; alternariol monomethyl ether; altenuene; tentoxin; tenuazonic acid; beauvericin; enniatin A, A1, B, and B1)	UHPLC-MS/MS	SPE	Methanol, acetonitrile, formic acid, ammonium format	0.01–0.2 ng/mL	1 mL	[31]
4	Citrinin, dihydrocitrinone, deoxynivalenol, fumonisin B1, T-2 toxin, HT-2 toxin, ochratoxin A, 2′R-ochratoxin A, ochratoxin α, tenuazonic acid, allo-tenuazonic acid, zearalenone, zearalanone, α-zearalenol, and β-zearalenol	HPLC-MS/MS	Dried spot	Methanol, acetonitrile, formic acid, ammonium bicarbonate	0.013–4.5 ng/mL	550 µL	[32]
5	Aflatoxin B1, aflatoxin B2, aflatoxin G1, aflatoxin G2, aflatoxin M1, zearalenone, ochratoxin A, zearalenone, alternariol monomethyl ether, sterigmatocystin, citrinin	UHPLC-MS/MS	Dilute-and-shoot	Acetonitrile, formic acid	LOQ 0.005–0.5 ng/mL	800 µL	[33]
6	Enniatins A, A1, B and B1, beauvericin	UHPLC-HRMS	DMSPE	Methanol, formic acid, ammonia solution	0.1–0.3 ng/mL	30 mL	[34]
7	Aflatoxin B2, aflatoxin, ochratoxin A, ochratoxin B, zearalenone, α-zearalenol	LC-Q-TOF-MS	QuEChERS	Acetonitrile, formic acid, MgSO_4_	1.5–5 ng/mL	1 mL	[35]
8	Aflatoxin B1, aflatoxin B2, aflatoxin G1, aflatoxin G2, aflatoxin M1, ochratoxin A, ochratoxin B, ochratoxin C, fumonisin B1, fumonisin B2, fumonisin B3, deoxynivalenol, 3-acetyldeoxynivalenol, 15-acetyldeoxynivalenol, T-2 toxin, HT-2 toxin, sterigmatocystin, cyclopiazonic acid, beauvericin, enniatin A, enniatin A1, enniatin B, enniatin B1, alternariol, alternariol monomethyl ether, altenuene, tentoxin, tenuazonic acid	UPLC-MS/MS	SPE	Methanol, acetonitrile, formic acid	0.0001–1.0 ng/mL	1 mL	[36]
9	Aflatoxin B1, ochratoxin A, zearalenone, deoxynivalenol	UHPLC-MS/MS	SPE	Methanol, acetonitrile, acetic acid, sodium acetate	0.08–6.6 ng/mL	3 mL	[37]
10	Aflatoxins B1 and M1, aflatoxin B1-N7-guanine adduct	UHPLC-HRMS	Dilute and shoot	Methanol, acetonitrile, formic acid, ammonium format	0.8–40 pg/mL	100 µL	[38]
11	Aflatoxins (aflatoxins B1, B2, G1, G2, M1), ochratoxin A, free ochratoxin α, gliotoxin, citrinin, dihydrocitrinone	HPLC-MS/MS	SPE	Methanol, ammonium acetate, acetic acid, hydrochloric acid	0.004–0.5 ng/mL	4 mL	[39]
12	Deoxynivalenol, ochratoxin A, zearalenone, α-zearalenol, de-epoxy deoxynivalenol, zearalenone-14 glucoside, α-zearalenol-14 glucoside	LC-Q-TOF-MS	Salting-out LLE	Methanol, acetonitrile, formic acid, ammonium acetate buffer, sodium chloride, C18 sorbent	0.33–1.5 ng/mL	1 mL	[40]

LC-Q-TOF-MS: liquid chromatography–quadrupole time-of-flight mass spectrometry. UHPLC-MS/MS: ultra-high-performance liquid chromatography–tandem mass spectrometry. UHPLC-HRMS: ultra-high-performance liquid chromatography–liquid chromatography–high-resolution mass spectrometry. SPE: solid-phase extraction. DMSPE: dispersive magnetic solid-phase extraction. QuEChERS: Quick, Easy, Cheap, Effective, Rugged, and Safe. LLE: liquid–liquid extraction.

**Table 2:** Assessment of LC methods using AGREE, MoGAPI, CACI, BAGI, and IMOGI metrics.

Method	AGREE	MoGAPI	CACI	BAGI	IMOGI
1 [29]	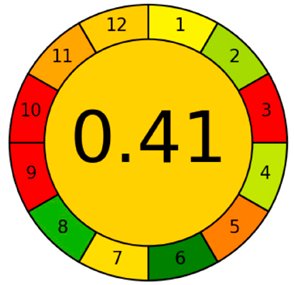	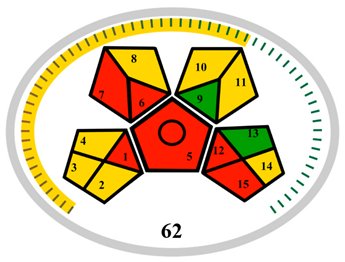	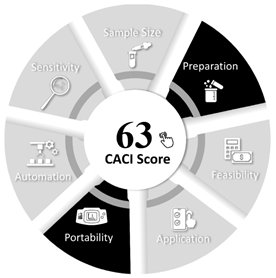	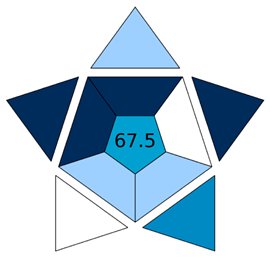	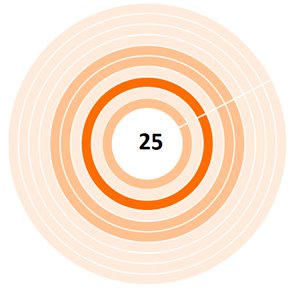
2 [30]	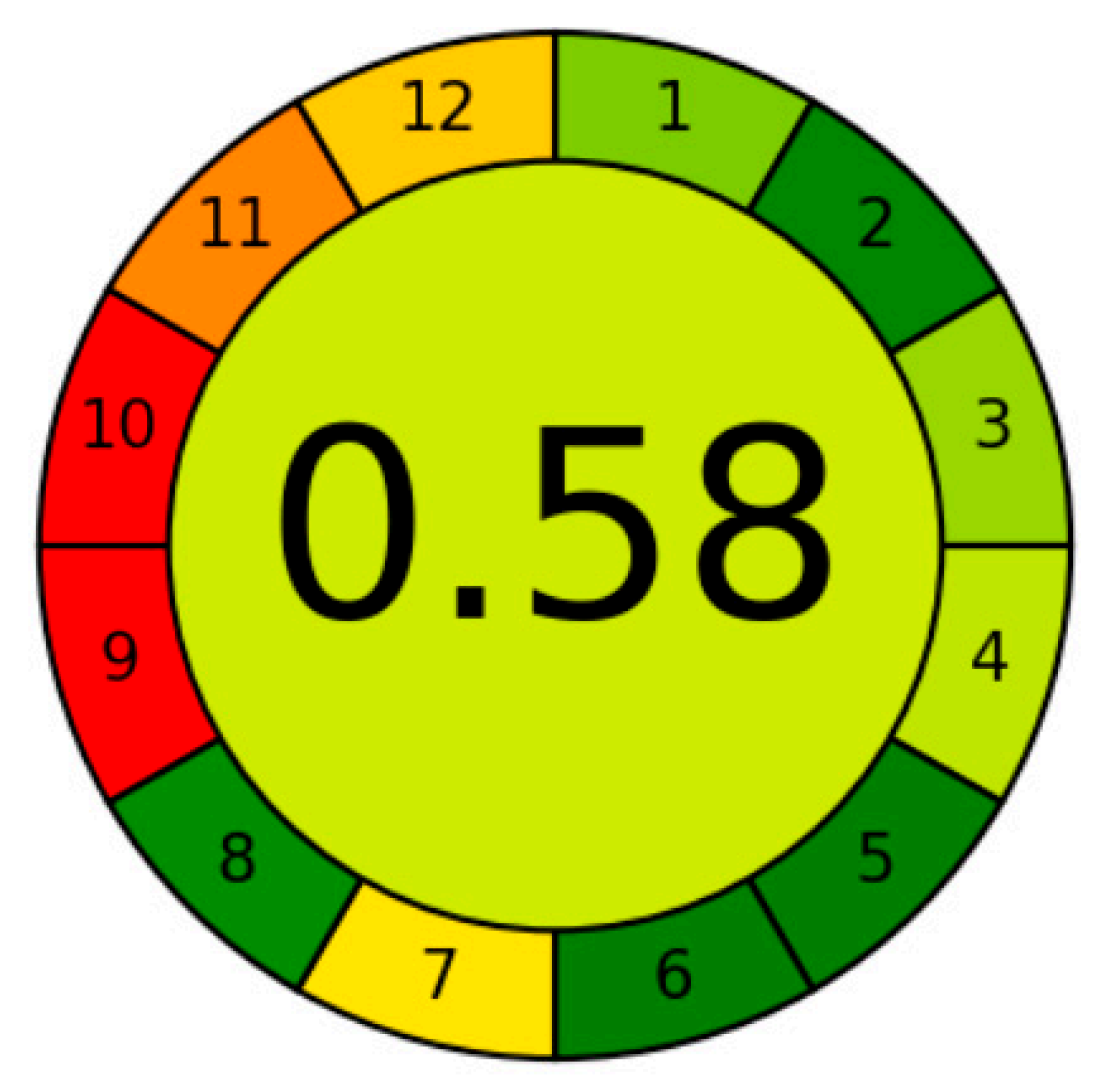	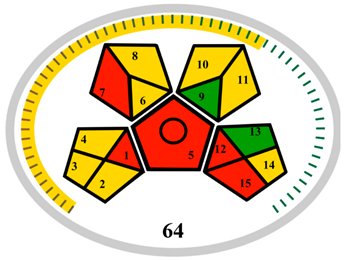	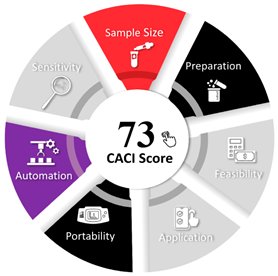	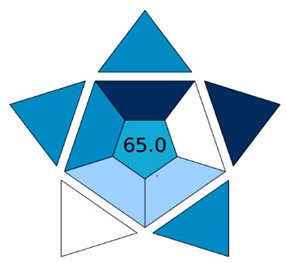	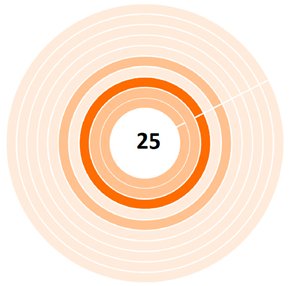
3 [31]	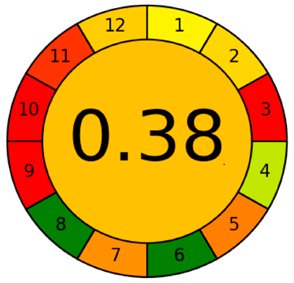	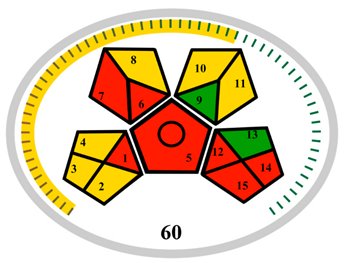	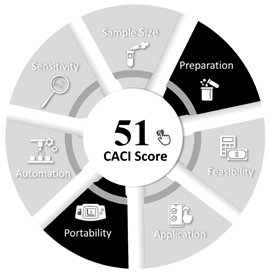	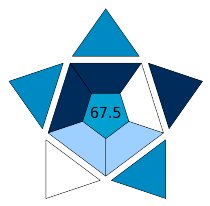	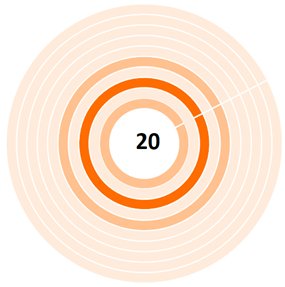
4 [32]	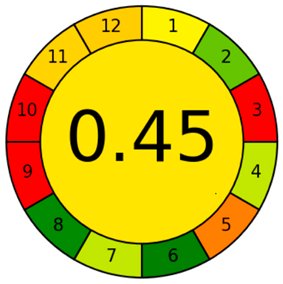	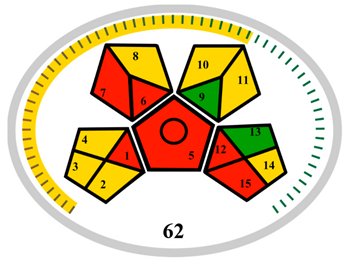	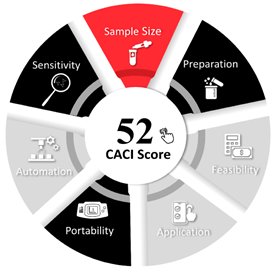	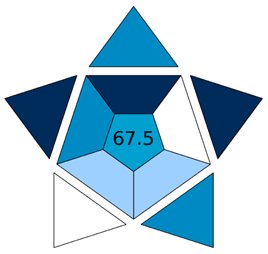	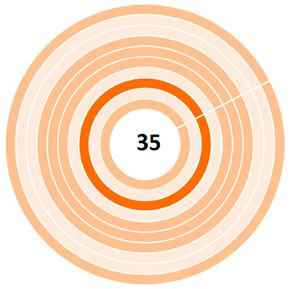
5 [33]	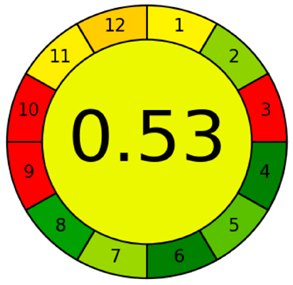	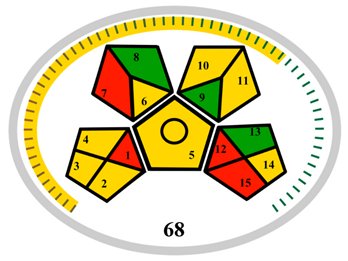	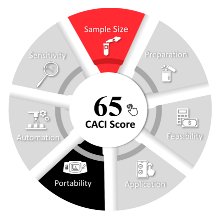	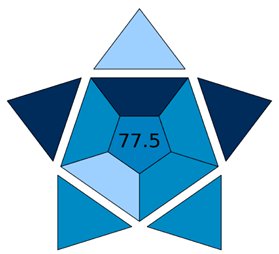	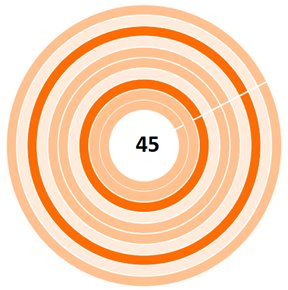
6 [34]	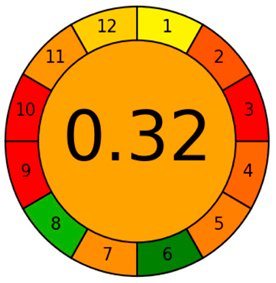	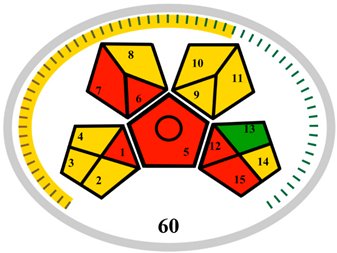	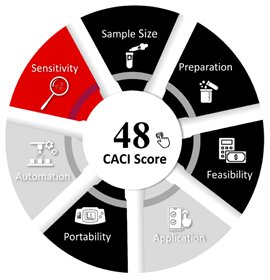	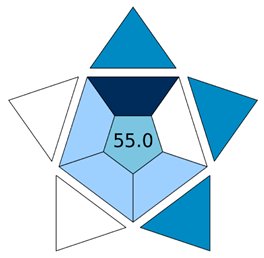	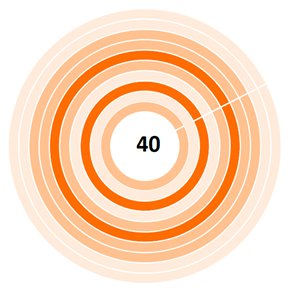
7 [35]	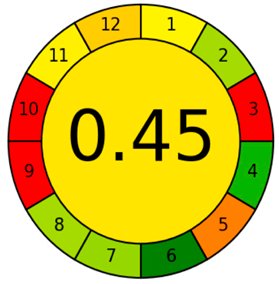	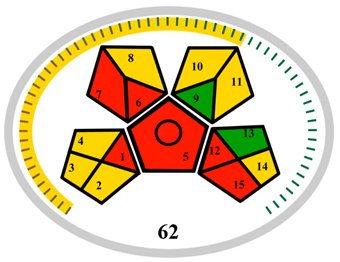	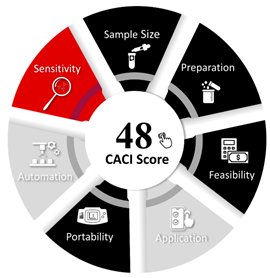	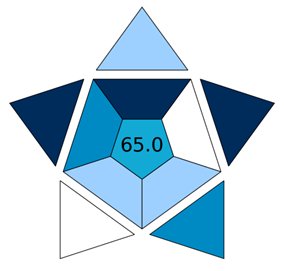	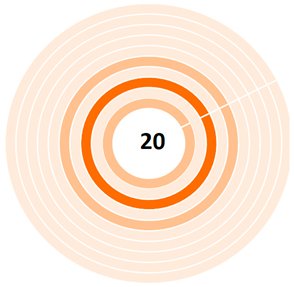
8 [36]	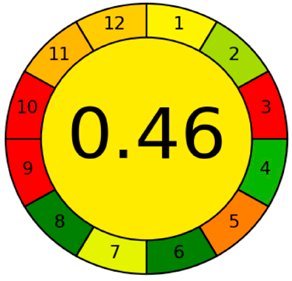	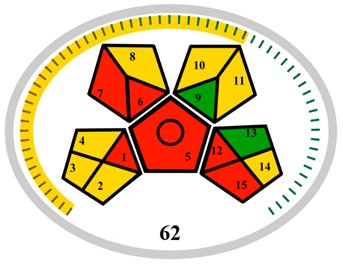	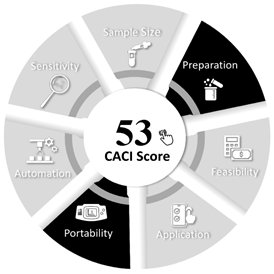	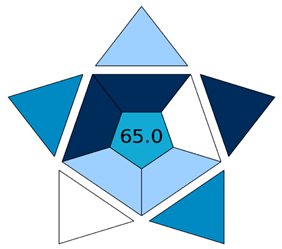	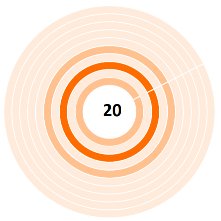
9 [37]	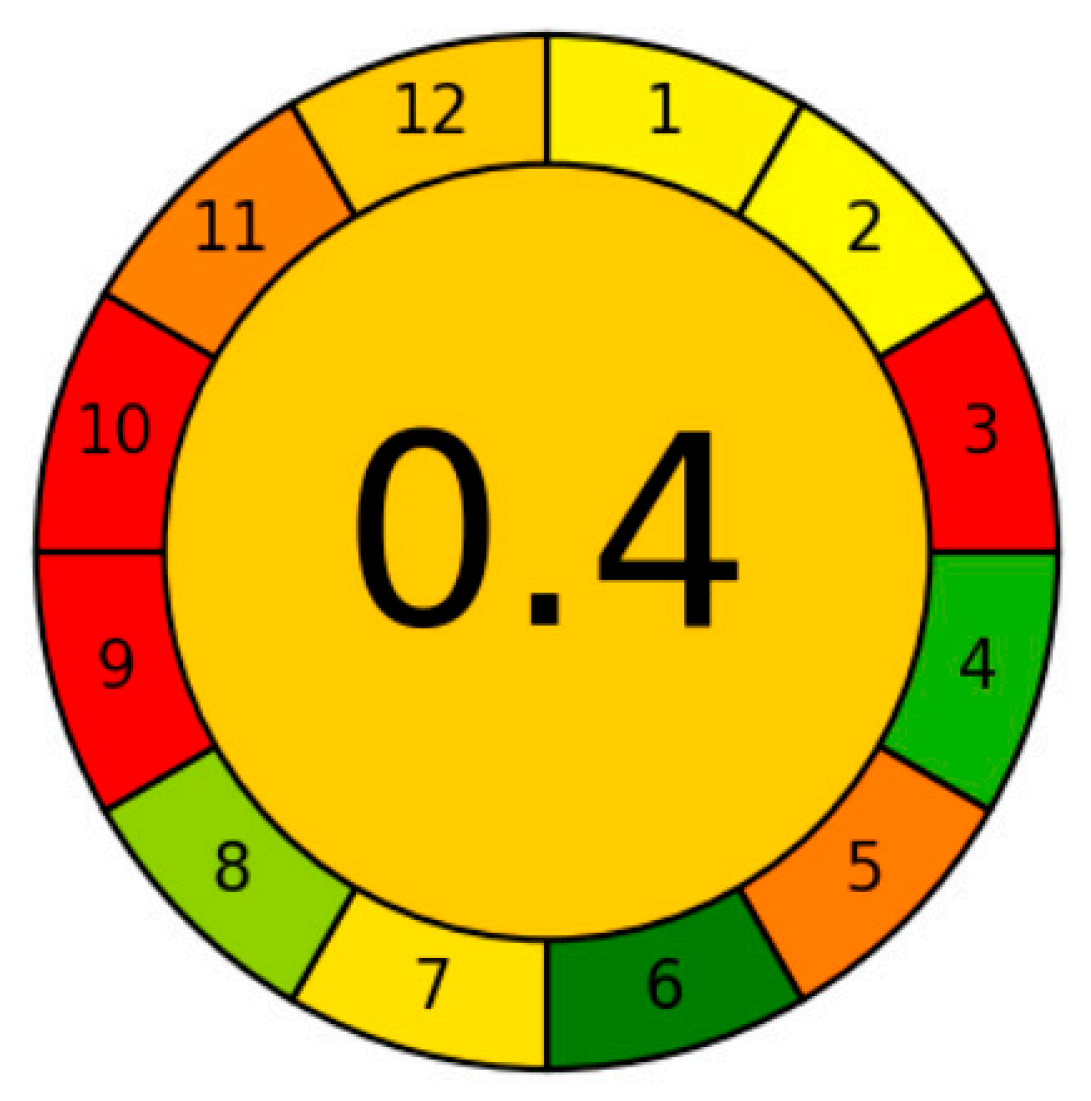	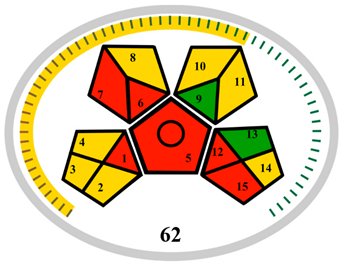	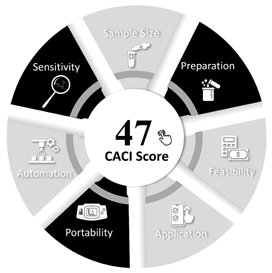	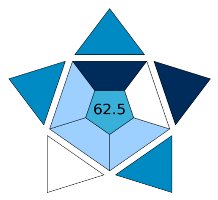	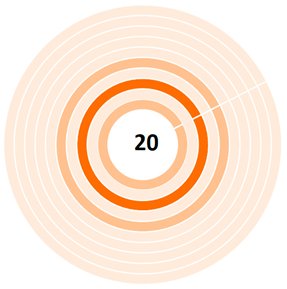
10 [38]	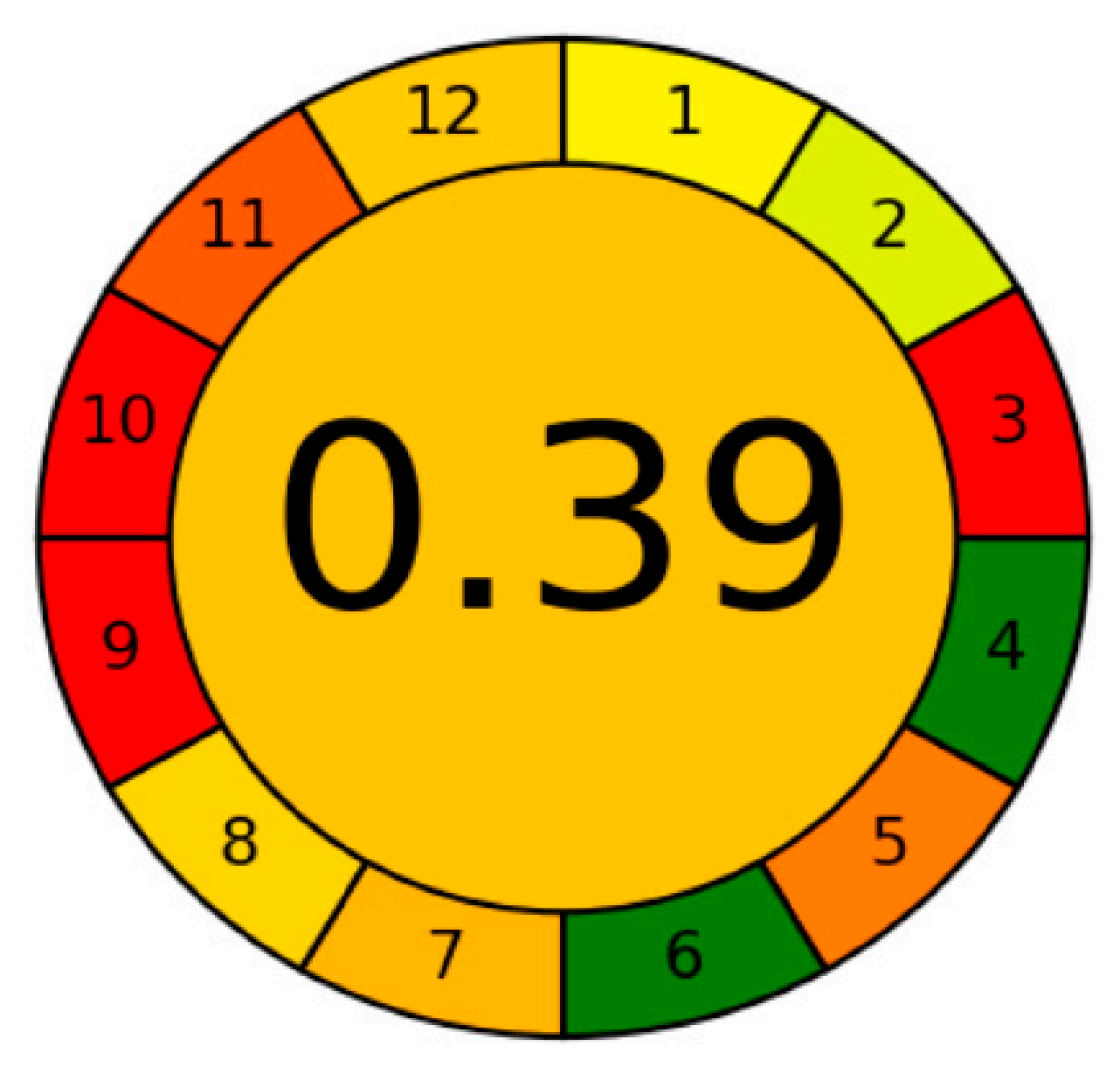	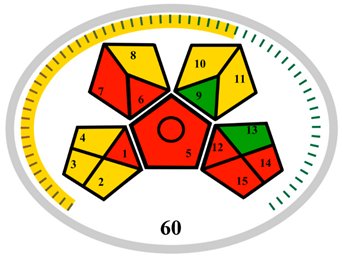	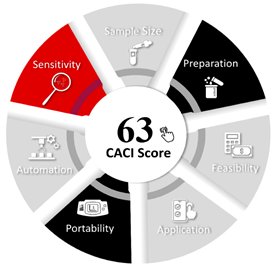	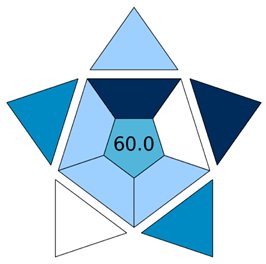	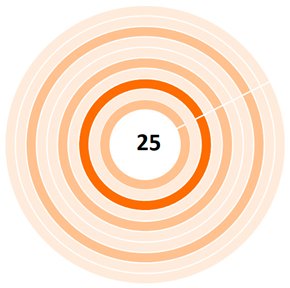
11 [39]	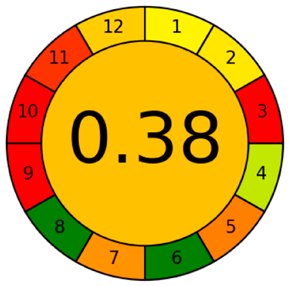	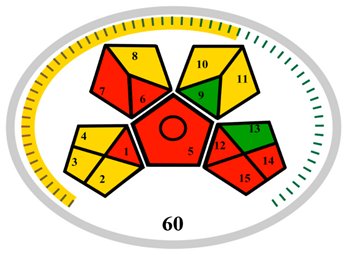	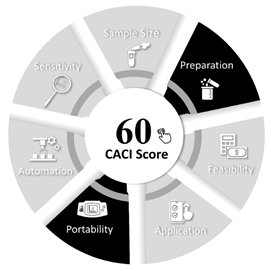	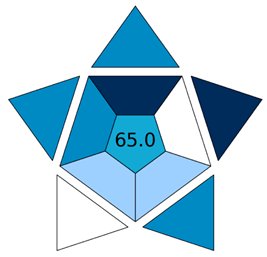	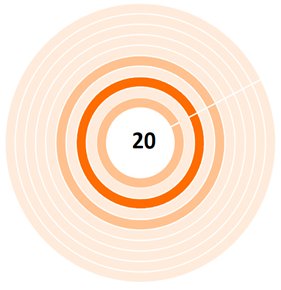
12 [40]	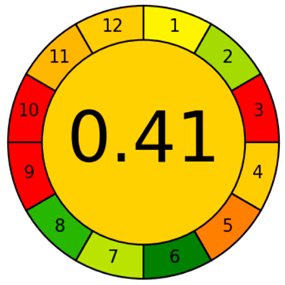	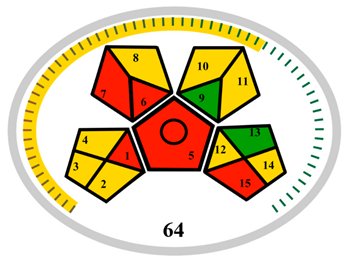	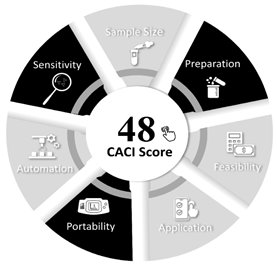	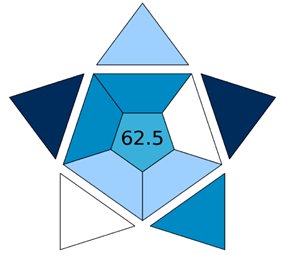	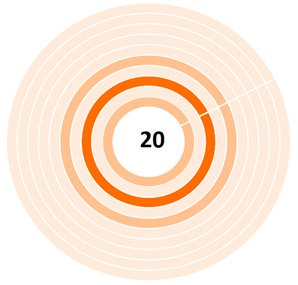

## Data Availability

The original contributions presented in this study are included in the article. Further inquiries can be directed to the corresponding author.
